# The efficacy and safety of electroacupuncture for diabetic peripheral neuropathy: A protocol for a systematic review and meta-analysis

**DOI:** 10.1371/journal.pone.0302228

**Published:** 2024-04-25

**Authors:** Jiawei Wang, Yajun Zhang, Qiqi Wu, Zhiyuan Bian, Ning Luo, Jing Sun, Binyan Yu, Jianqiao Fang

**Affiliations:** 1 The Third Clinical College of Zhejiang Chinese Medical University, Hangzhou, Zhejiang, China; 2 Department of Acupuncture, Moxibustion and Massage, Wenzhou Central Hospital, Wenzhou, Zhejiang, China; 3 Department of Acupuncture and Moxibustion, The Third Affiliated Hospital of Zhejiang Chinese Medical University, Hangzhou, Zhejiang, China; 4 Department of Acupuncture and Moxibustion, The First Affiliated Hospital of Zhejiang Chinese Medical University, Hangzhou, Zhejiang, China; Baylor College of Medicine, UNITED STATES

## Abstract

**Background:**

Diabetic peripheral neuropathy (DPN) is a chronic complication of diabetes mellitus, which is the most common neuropathy worldwide. Owing to the inadequacies of existing treatment methods, managing DPN remains a significant challenge. Studies suggest that electroacupuncture (EA) could potentially serve as a beneficial alternative treatment for this condition. Nevertheless, there is still inadequate proof of its therapeutic effectiveness and safety. As a result, the goal of this protocol is to methodically compile the data pertaining to the effectiveness and security of EA in the management of DPN.

**Methods:**

To find appropriate randomized controlled trials (RCTs), nine reliable databases in the English and Chinese languages will be examined. RevMan5.3 will be used to combine the retrieved data and perform meta-analyses. The methodological quality of the included RCTs will be evaluated using the Cochrane Risk of Bias Assessment 2.0 tool. The Grades of Recommendations, Assessment, Development, and Evaluation (GRADE) system will be utilized to evaluate the degree of strength and certainty of the evidence. We will also perform publication bias, sensitivity and subgroup analyses.

**Discussion:**

This protocol describes the intended scope and approach for a forthcoming systematic review and meta-analysis that will inform therapeutic decision-making by offering current information on the efficacy and safety of EA in the treatment of DPN. The results of the study will help standardize strategies for EA in the treatment of DPN.

## Introduction

Diabetic peripheral neuropathy (DPN) is one of the most serious, frequent and complex side effects of diabetes mellitus [1]. A minimum of 200 million people worldwide are believed to have neuropathy issues related to pre-diabetes and diabetes mellitus, with 316 million and 387 million affected, respectively, and develop some form of neuropathy during their lifetime [2]. According to epidemiological data, the prevalence of neuropathy has been reported to be as high as 30% in the diabetic population [3]. Of these, approximately 15% of diabetic patients suffer from diabetic foot ulcers, with more than 15% needing amputations [4]. The treatment requirements of DPN patients have received more attention in an effort to successfully address these issues.

DPN affects different parts of the nervous system and triggers a series of symptoms involving different types of pathologic mechanisms [5]. In addition to the typical stocking-glove distribution of numbness, tingling, pain, or weakness that begins at the distal ends of the limbs, individuals diagnosed with DPN may also experience significant muscular atrophy, deformities, limb paralysis, etc. Being a chronic and disabling disease, DPN causes great physical, emotional, and social burdens, leading to a considerable depletion of both healthcare and economic resources [6].

Despite the increased understanding of DPN unfolding in recent years, there is still a dearth of research on DPN, the clinical diagnosis and treatment of DPN are greatly inadequate. At present, for DPN patients with poor blood glucose control, treatment is mainly based on hypoglycemic drugs (biguanides, second-generation sulfonylureas, etc.) and insulin therapy, adding diet control with exercise and other non-drug interventions [7]. However, previous meta-analyses have also reported that symptoms in DPN patients are less affected by blood glucose, indicating that glycemic management measures alone might not be adequate to stop DPN from developing or worsening [8]. As the breadth and depth of global research increase, the selection of drug types for DPN treatment has also become more diversified, such as nutritive neurotics, antioxidants, microcirculation improvement, analgesics and other drugs as the mainstay. Provide an instance, several reviews have already been published indicating that combination therapies such as methylcobalamin were more effective than monotherapies for patients with DPN [9], but it has not been demonstrated to have a targeted improvement of peripheral nerve function [10, 11]. Other drugs like Alpha-lipoic acid, angiotensin-converting enzyme inhibitors, aspirin and opioids are reported to lessen DPN symptoms. Nevertheless, their application in clinical practice is frequently restricted, especially in the sickest patients who require them the most, leaving patients with few alternatives for long-term pharmaceutical management [12–17]. The side effects, long-term usage concerns, and uncertain efficacy of these medications limit their broader application [18–20], thus heightening the demand for new, alternative treatments. Consequently, there is a growing interest in acupuncture-related therapies as potential alternatives for managing DPN.

Acupuncture, including electroacupuncture (EA), has shown positive efficacy in treating Diabetic Peripheral Neuropathy (DPN). Studies indicate it not only reduces DPN symptoms but also aids in glycemic control [21–24]. EA is an extension technique based on acupuncture that combines electrical stimulation and traditional acupuncture that can be adjusted to different waveforms, frequencies, stimulation intensities and duration according to personal needs, thus improving efficacy and making it more accessible to DPN patients [25]. A previous animal experiment conducted by our research group showed that EA at 2 and 100Hz attenuated pain in type 2 diabetic neuropathic pain rats [26]. Because the majority of the prior research used animal and cell models for pre-clinical investigations or only contained a small number of clinical samples, the therapeutic effect of clinical EA on DPN remained unclear [27–29]. To date, several randomized controlled trials (RCTs) assessing the efficacy and safety of EA have been conducted, demonstrating the benefits of EA in relation to DPN symptom relief [30–32]. For example, a multicenter RCT of acupuncture published in 2018 demonstrated that the efficacy of EA for DPN was significantly superior to that of the treatments for the control group after 8-weeks of treatment [30]. Notwithstanding an earlier systematic review and meta-analysis that sought to assess the data pertaining to the impact of EA on DPN, the conclusions of that study did not have rigorous, conclusive data to support the efficacy of EA for the treatment of DPN, thus failing to obtain substantial evidence that EA therapy can be used as a recommended therapy for patients with DPN. Moreover, it is also worth noting that the total number of RCTs included in the above-mentioned previous systematic review and meta-analysis was only four, and all of them were published in Chinese journals, with a retrieval time span of only 3 months [33]. Current clinical studies generally support EA’s efficacy, but a lack of comprehensive evaluation and safety data persists. Ongoing, well-designed RCTs, such as one by Li et al., are expected to provide more definitive evidence on EA’s efficacy and safety, using medical imaging as primary outcome metrics [34].

In summary, as global research deepens, there is an urgent need to synthesize rigorous and innovative data supporting the safety and effectiveness of EA in the management of DPN.

### Objectives

The current protocol for a subsequent systematic review and meta-analysis, which aims to synthetize the current evidence regarding the efficacy and safety of EA in the treatment of DPN.

## Methods and analysis

To increase the openness and transparency of this study, the Preferred Reporting Items Statement for Systematic Reviews and Meta-Analysis Protocols (PRISMA-P) guidelines are strictly adhered to and the study was registered on the Prospero International Prospective Registry for Systematic Reviews under the number CRD42023491269 [35] (S1 Table).

### Eligibility criteria

The PICOS component as the methodology used to conduct the systematic review and meta-analysis will be applied to establish trial eligibility.

### Study designs

Our systematic review and meta-analysis will only consider RCTs that are intended to investigate the effectiveness of EA treatment for DPN. Only Chinese and English will be included. In addition, for crossover RCTs that were enrolled by reference to related studies, we will only consider preliminary data from the first two crossover groups in the meta-analysis [36]. Non-RCTs, case series, reviews, and other research types that are not eligible will not be taken into consideration in this systematic review and meta-analysis.

### Participants

Selected participants are patients with a definitive clinical diagnosis of DPN: 1) Diagnosed with diabetes mellitus by a clinician. 2) In the case of arms and legs (at least in the double lower limbs) with sensory disturbance and/or persistent pain; electromyography detects lower motor and sensory nerve conduction, prolonged latency, decreased amplitude, and other electrophysiologic abnormalities; Positive signs on neurologic examination; Michigan Neuropathy Screening Instrument questionnaire score of 7 or greater [37]. Patients will be eligible regardless of age, sex, country, race, or case origin.

### Interventions

The main intervention for the treatment group is EA, with no requirements for the duration, waveform, or intensity of EA use. The treatment group can use EA alone or in combination with medication in the control group. To reduce heterogeneity, manual acupuncture, warm needle acupuncture, auricular acupuncture, laser acupuncture, transcutaneous electrical acupoint stimulation and acupressure are excluded.

### Comparators

1. Treated with guideline-recommended diabetes medication (eg, biguanides, second-generation sulfonylureas, insulin products) [7] or medication that has been clinically proven to be effective for DPN symptoms [9, 12, 13, 16].
2. Placebo controls: sham acupuncture, placebo medication.
3. Waiting list, no intervention.

### Outcomes

After referring to the relevant references [38, 39] to determine the results relevant to our method. Accordingly, at least one of the following results should be reported in qualified trials.

### Primary outcomes

1. Sensory nerve conduction velocity (SNCV)
2. Motor nerve conduction velocity (MNCV)

(DPN affects the conduction velocities of both motor and sensory nerves, making them useful markers for severity of illness. Particular neurological conditions include those involving the ulnar, median, and sural sensory nerves, as well as the ulnar, peroneal, and tibial motor nerves, etc.)

### Secondary outcomes

1. Michigan Diabetic Neuropathy Score (MDNS)
2. New Injury Severity Score (NISS -LL) score
3. Toronto Clinical Scoring System (TCSS)
4. Blood biochemistry, Glucose indices (eg, FBG, 2hPG, and HbA1c)
5. Adverse events.

### Information sources

Each potentially qualifying RCT will be thoroughly searched throughout the following nine databases, including 5 English databases (Web of Science, PubMed, Physiotherapy Evidence Database (PEDro), Cochrane Central Register of Controlled Trials, EMBASE) and 4 Chinese databases (Chinese National Knowledge Infrastructure, VIP Database for Chinese Technical Periodicals, Wangfang database, Chinese Biomedical Literature Database), spanning from the beginning of the data to December 2023.

### Search strategy

The retrieval approach includes three basic elements: intervention (eg, electric acupuncture, electroacupuncture, electrical acupuncture, EA); participants (eg, diabetes mellitus, diabetic neuropathy, diabetic peripheral neuropathy, DPN, peripheral nervous system diseases); study type (randomized controlled trial, RCT). We will search the English database either separately or in combination using the aforementioned English-language search phrases. These words will be replaced by Chinese phrases with the same meaning in Chinese databases. In order to guarantee thorough database coverage and take into account a broad spectrum of potential matches, we will combine topic words (such as medical subject headings [MeSH]) with free-text phrases. To obtain higher sensitivity, the MeSH terms can be replaced with appropriate subject terms and paired with the proper free-text term searches. Depending on the query requirements of different databases, search strategy can be adjusted. The search strategy in the PubMed, Embase, and Web of Science databases are shown in Tables 1–3.

**Table 1:** Search strategies for PubMed.

No.	Search Items
#1	Randomized controlled trial [pt]
#2	Controlled clinical trial [pt]
#3	Randomized OR Randomised [Title/Abstract]
#4	Clinical trials [MeSH]
#5	Randomly [Title/Abstract]
#6	Trial [Title/Abstract]
#7	#1 OR #2 OR #3 OR #4 OR #5 OR #6
#8	Diabetic peripheral neuropathy [MeSH Terms]
#9	(Diabetes mellitus OR Diabetic neuropathy OR DPN OR Diabetic OR Diabetics OR Peripheral nervous system diseases OR Diabetic neuropathy pain OR Diabetes Complications OR Diabetes insipidus OR Macro and micro-vascular diabetic complication OR Diabetic polyneuropathy) [Title/Abstract]
#10	#8 OR #9
#11	Electroacupuncture [MeSH Terms]
#12	(EA OR Electric acupuncture OR Electro-acupuncture OR Galvanoacupuncture OR Acupuncture) [Title/Abstract]
#13	#11 OR #12
#14	#7 AND #10 AND #13

**Table 2:** Search strategies for Embase.

No.	Search Items
#1	Randomized controlled trial:ti,ab,kw
#2	Controlled clinical trial:ti,ab,kw
#3	Randomized OR Randomised:ti,ab,kw
#4	Clinical trials/exp
#5	Randomly:ti,ab,kw
#6	Trial:ti,ab,kw
#7	#1 OR #2 OR #3 OR #4 OR #5 OR #6
#8	Diabetic peripheral neuropathy/exp
#9	(Diabetes mellitus OR Diabetic neuropathy OR DPN OR Diabetic OR Diabetics OR Peripheral nervous system diseases OR Diabetic neuropathy pain OR Diabetes Complications OR Diabetes insipidus OR Macro and micro-vascular diabetic complication OR Diabetic polyneuropathy):ti,ab,kw
#10	#8 OR #9
#11	Electroacupuncture/exp
#12	(EA OR Electric acupuncture OR Electro-acupuncture OR Galvanoacupuncture OR Acupuncture):ti,ab,kw
#13	#11 OR #12
#14	#7 AND #10 AND #13

**Table 3:** Search strategies for Web of Science.

No.	Search Items
#1	TS = (“Randomized controlled trial” OR “Controlled clinical trial” OR “Randomized” OR “Randomised” OR “Clinical trials” OR “Randomly” OR “Trial”)
#2	TS = (“Diabetic peripheral neuropathy” OR “Diabetes mellitus” OR “Diabetic neuropathy” OR “DPN” OR “Diabetic” OR “Diabetics” OR “Peripheral nervous system diseases” OR “Diabetic neuropathy pain” OR “Diabetes Complications” OR “Diabetes insipidus” OR “Macro and micro-vascular diabetic complication” OR “Diabetic polyneuropathy”)
#3	TS = (“Electroacupuncture” OR “EA” OR “Electric acupuncture” OR “Electro-acupuncture” OR “Galvanoacupuncture” OR “Acupuncture”)
#4	#1 AND #2 AND #3

Also, we will scrutinize the references of systematic reviews and meta-analyses pertaining to DPN. For future updates of this review, we will search the Chinese Clinical Trial Registry (http://www.chictr.org.cn), the World Health Organization’s International Clinical Trials Registry Platform (https://www.who.int/clinical-trials-registry-platform), and the Clinical Trials Registry (https://clinicaltrials.gov/) to avoid missing ongoing eligible RCTs.

### Study selection process

NoteExpress (V3.X, Aegean Sea software company, Beijing, China) will be used to record the retrieved records collected from all databases to delete duplicate studies. During the initial screening of literature, two independent reviewers (J.W) and (Q.W) will examine the titles and abstracts of publications through the preceding inclusion/exclusion criteria to identify and select any eligible RCTs. To further verify the validity of the included literature, a full-text evaluation of potentially eligible articles will be performed. If two reviewers disagree on an included publication, a third referee (J.F) is required to adjudicate the discussion. S1 Fig provides a summary of the study’s selection procedure.

### Data extraction and data items

To collect relevant information, after completing the inclusion of all eligible RCTs, two impartial reviewers (Z.B and N.L) will use pre-defined spreadsheets (as shown in S2 Table) to compile information on author names, publication years, research designs, participant characteristics, sample sizes, and the number of sessions, duration, and outcome measures of the interventions in the experimental group and the control arm. The means and standard deviations (SDs) for continuous results will be obtained. If continuous results are given in various formats (such as mean [95% CI], median [interquartile range], etc.), they will be transformed into means (SDs) using the procedures suggested in the Cochrane Handbook for the Assessment of Intervention Systems [40]. The total number of individuals in each research group as well as the number of responders will be acquired with regard to dichotomous outcomes. In the event that the original publication is lacking in the necessary related data, the reviewers will contact the authors via email to compensate for the missing information. Any disagreement between reviewers during the process will be adjudicated through the senior reviewer (J.F).

Prior to adequately obtaining data from every research project which is included, we will use the Kappa coefficients will assess inter-rater consistency regarding the precision of data extraction by extracting data from 10 randomly selected studies. Evaluators will receive further training in data extraction once the a deficiency in accuracy and uniformity between raters.

### Methodological quality assessment

The two independent raters (J.S and B.Y) will evaluate the methodological quality of all included RCTs in accordance with the Cochrane recommended risk of bias 2.0 (RoB 2.0) tool [41]. Every included study’s RoB will be graded based on five major criteria: 1) method of randomization; 2) departure from the planned intervention; 3) incomplete results information; 4) measurement of outcomes; and 5) selection of results reported. Three ratings will be granted to each domain: “low”, “unclear”, or “high”. In addition, reviewers will provide an overall ROB for each trial of “low” (indicating a low ROB for all domains), “unclear” (indicating some concerns in one or more domains), or “high” (indicating high ROB in one or more domains, or some concerns in a different domain) ratings. The senior arbitrator (J.F) will mediate any disagreements between reviewers through discussions.

### Data synthesis and statistical analysis

The Cochrane Handbook for Systematic Reviews of Interventions and the Review Manager (RevMan) software (Version 5.30, Cochrane Collaboration, Oxford, UK) will be consulted in conducting the risk of bias assessment of each involved publication. Relative risk (RR) will be utilized for presenting dichotomous data and standardized mean difference (SMD) with 95% confidence intervals (SMD) will be performed to describe continuous variables. Statistical heterogeneity between trials will be assessed using I^2^ and categorized into three classes: low (I^2^ < 50%), medium (I^2^ between 50%-75%), and high (I^2^ > 75%). Both modeling approaches, a fixed-effect model or a random-effects model, estimate a single effect size of interest. The fixed-effect meta-analysis assumes that all studies share a single common effect and, as a result, all of the variance in observed effect sizes is attributable to sampling error. The random-effects meta-analysis estimates the mean of a distribution of effects, thus assuming that study effect sizes vary from one study to the next. Under this model, variance in observed effect sizes is attributable to both sampling error (within-study variance) and statistical heterogeneity (between-study variance). The most popular meta-analyses involve using a weighted average to combine the study-level effect sizes. Different effect models will be chosen for different heterogeneities to make the meta-analysis results more robust and reliable. Using a fixed-effects model, heterogeneity will be rated as low; a random-effects model for combined analyses will be used to rate heterogeneity as medium; and a discussion with the review panel to explore clinical or methodologic heterogeneity will be used to rate heterogeneity as high. Narrative analyses will be performed when meta-analysis is not possible.

### Subgroup analysis

The following criteria will be considered in our subgroup analysis if backed up by a sufficient number of samples from subgroups and other feasible conditions.

1. Different waveforms of EA (continuous wave, sparse-dense wave, intermittent wave).
2. Duration and severity of disease.
3. Type of control arm (eg, active control, sham control).
4. Various assessment points according to the main results.

### Assessment of the quality of evidence

The quality and reliability of the evidence will be evaluated according to Cochrane’s recommended Grading of Recommendations, Assessment, Development and Evaluation (GRADE) approach. The evidence’s degree of certainty and strength will be summarized by using general “confidence in evidence” ratings, which are categorized as high, medium, low, and very low [42].

### Publication bias

Once the meta-analysis carried ten or more RCTs, Egger’s test and funnel plots will be used to assess for publication bias.

### Ethical consideration

This project doesn’t involve ethical approval given its outcomes will be published in a peer-reviewed publication and personal data from participants will not be included in the systematic review and meta-analysis.

## Discussion

Recently, the prevalence of DPN has been gradually increasing worldwide. At the same time, medical professionals face the formidable challenge of managing DPN with limited alternatives and insufficient knowledge about the causative mechanisms.

There is still uncertainty regarding the pathophysiology of DPN and the risk factors that are linked to it. Patients with diabetes mellitus often experience high glucose and fat levels. These conditions, along with neuronal inflammation, oxidative stress, mitochondrial dysfunction, and apoptosis caused by microangiopathy, are key factors contributing to the high incidence of DPN morbidity [43]. As an essential component of acupuncture TCM, EA has been extensively applied in clinical settings. EA improves the excitability of the nerve, which can inhibit ectopic discharges from nerve fibers and promote the repair and regeneration of injured peripheral nerves. The current study’s outcomes support the growing body of research demonstrating that EA is a viable and effective treatment option for DPN [30, 44]. However, the exact mechanisms by which this occurs are not fully understood and more evidence is needed to make EA treatment clinically acceptable. One of the primary diagnostic markers of DPN is nerve conduction velocity (NCV), which is constantly used to evaluate nerve function [2]. Research on humans and animals equally demonstrated that EA facilitates the preferential re-innervation of motor and sensory neurons [45–47]. For instance, there are animal experiments illustrating that EA could be connected to metabolic control and the secondary effect on the GLO/AGE/RAGE axis since it plays a part in treating T2DM-induced DPN [48]. Currently, most clinical trials and animal studies have demonstrated a positive effect of EA for the treatment of DPN. However, more detailed mechanisms of EA for DPN and the effects of different EA waveforms on efficacy need to be further investigated and demonstrated.

Following is a discussion of the merits of this study. First of all, a previous meta-analysis published in Chinese showed the therapeutic effect of EA on DPN was significantly better than that of Western medicine. And all the included RCTs were statistically significant (P < 0.05). The following conclusions were drawn: Compared to purely Western medicine treatment, EA not only effectively relieved patients’ clinical symptoms such as pain, numbness, and chills, but also improved patients’ nerve conduction velocity and blood biochemical indexes. But the included RCT studies are conducted only in China with small sample sizes and lacking a large span of search time, resulting in low-quality evidence [33]. Besides, compared to acupuncture-related trials conducted in Western nations, the percentage of positive findings in Chinese trials is significantly higher [49]. Therefore, we believe that the number of RCTs with higher-quality evidence has been increasing in recent years, the latest evidence of EA for DPN research needs to be constantly updated. A rigorous systematic review and meta-analysis on the use of EA in the treatment of DPN is still lacking, despite several RCTs having demonstrated EA’s effectiveness in treating DPN [30–32]. In the above context, it is necessary to conduct a systematic review and meta-analysis to comprehensively collate and summarise the evidence on the efficacy and safety of EA in the treatment of DPN. When the research’s future results are demonstrated, we will enrich an effective therapy for DPN and further clarify the applicable population, hence offering clinical decision-making grounded in evidence-based medicine.

Second, the requirements established by Preferred Reporting Items for Systematic Reviews and Meta-Analyses (PRISMA) shall be adhered to highly rigorously in this review [35]. We will search 9 mainstream English and Chinese databases for relevant studies with a comprehensive and systematic search, applying strict inclusion criteria for investigational clinical trials and extending the search time span from the start of the data to December 2023.

Third, trying to avoid future performance bias, we will follow the existing guidelines included in the Cochrane Collaboration Handbook, which will go a long way towards standardizing the meta-analysis process and ensuring the quality of subsequent meta-analysis results in advance. Where possible, we will also perform sensitivity analyses, publication bias, and subgroup analyses to enhance the integrity of the protocol.

Of course, this study has certain innovation and reference, but some problems need to be explained. First, by limiting the results to English and Chinese databases, there might be a linguistic bias that prevents qualifying RCTs published in other languages from being discovered. Second, RCT studies have varying degrees of quality and the results of research outcome markers vary. This heterogeneity in results may lead to difficulties in interpreting findings and making a recommendation. Third, distinct EA features (current frequency, acupoints, waveform, and retaining time, etc.) will likewise pose a great risk of heterogeneity in the combined outcomes. Fourth, we expect that most of the included RCT results will come from China, as acupuncture is one of the traditional Chinese treatment modalities. In trials pertaining to acupuncture, the percentage of satisfactory outcomes in China is significantly higher than in Western nations. Characteristics of RCTs of acupuncture in China and the West are different. The Western focus on whether acupuncture is effective for a particular type of disease has focused on validating the specific efficacy induced by acupuncture. The implementation of patient blinding is mostly used. The majority of the trials found minimal difference in the effectiveness of acupuncture and placebo or sham acupuncture. In China, most of the published RCTs of acupuncture focus on the efficacy of acupuncture and are comparative studies of overall efficacy. These studies have demonstrated that a certain acupuncture method or a certain choice of acupoints is superior to conventional acupuncture treatment or conventional Western medical treatment, and most of them have positive results [49]. To address the risk of bias highlighted in the included studies, RCTs included in future studies need to fulfill the following conditions: 1) appropriate outcome indicators; 2) reasonable treatment protocols; 3) selection of a reasonable control group; and 4) setting access criteria for acupuncturists participating in the trial.

Taken together, this raises challenging questions for current research: whether DPN can be treated with EA and how to use it more efficiently.

## Conclusions

In summary, the protocol outlines the rationale and anticipated approach for a proposed systematic review and meta-analysis of data conducted to evaluate the efficacy and safety of EA for the treatment of DPN. The anticipated findings will provide evidence-based clinical judgments to assist physicians’ clinical decision-making and help patients make an informed choice about choosing this treatment.

## Supporting information

S1 Fig The PRISMA flow diagram of the study selection process. (TIF)

S1 Table PRISMA-P (Preferred Reporting Items for Systematic review and Meta-Analysis Protocols) 2015 checklist: Recommended items to address in a systematic review protocol. (DOC)

S2 Table The pre-defined electronic form to extract the characteristics of the included RCTs. (DOCX)
