# Supplementary figures and images for: The efficacy and safety of electroacupuncture for diabetic peripheral neuropathy: A protocol for a systematic review and meta-analysis
Source: PLoS One. 2024 Apr 25;19(4):e0302228. doi: 10.1371/journal.pone.0302228 (PMC11045088; doi:10.1371/journal.pone.0302228)

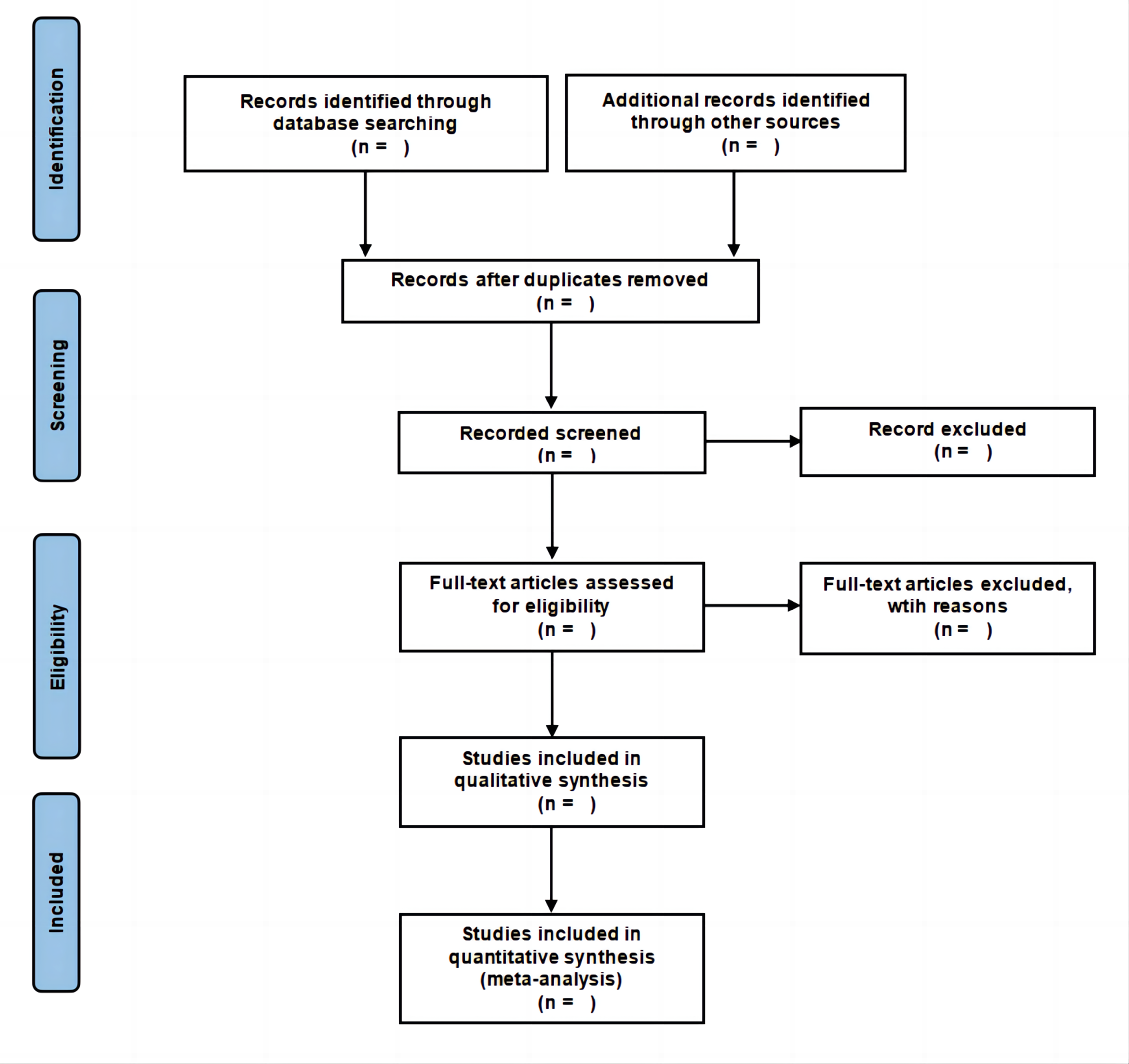

Supplement: S1 Fig — (TIF) [file pone.0302228.s001.tif]
