# Supplementary material for: The efficacy and safety of electroacupuncture for diabetic peripheral neuropathy: A protocol for a systematic review and meta-analysis
Source: PLoS One. 2024 Apr 25;19(4):e0302228. doi: 10.1371/journal.pone.0302228 (PMC11045088; doi:10.1371/journal.pone.0302228)
Supplement: S2 Table — (DOCX) [file pone.0302228.s003.docx]

**Supplementary Table 2. The pre-defined electronic form to extract the characteristics of the included RCTs**

**Sample Size**（**male/female**）

**Treatment protocol**

**The Duration of Outcome**

**Study Study design Intervention Control Intervention Control number of treatment and measurements**

**(author, group group group group treatment follow-up**

**year, sessions**

**country)**

|  |
| --- |
|  |
